# Proximal Tibiofibular Joint Dysfunction as a Cause of Persistent Knee Pain After Total Knee Arthroplasty: A Case Series and Literature Review

**DOI:** 10.7759/cureus.35367

**Published:** 2023-02-23

**Authors:** Volodymyr Toropchyn, Sanjeev Kumar

**Affiliations:** 1 Anesthesiology, University of Florida, Gainesville, USA

**Keywords:** tka, ultrasound guided injections, pain after total knee arthroplasty, persistent knee pain, ptfj, proximal tibiofibular joint

## Abstract

Total knee arthroplasty (TKA) is a common procedure for end-stage osteoarthritis of the tibiofemoral and patellafemoral joints. Despite a good outcome in many patients, persistent knee pain after TKA poses a significant challenge. Proximal tibiofibular joint (PTFJ) osteoarthritis has been seen as a rare cause of such pain. In this case series, we share our experience diagnosing PTFJ dysfunction and managing it with intra-articular ultrasound-guided injections. We demonstrate that PTFJ arthropathy may be a more common cause of chronic post-TKA pain than generally believed.

## Introduction

Total knee arthroplasty (TKA) is a common procedure for the treatment of end-stage osteoarthritis of the tibiofemoral and patellafemoral joints [1]. With the increasing rate of primary TKAs, the number of patients with TKA complications has seen a similar trend. Despite a good outcome for many patients, approximately 20% of patients experience chronic pain after TKA [2]. Overall, the diagnosis and treatment of the painful TKA can pose a significant challenge. Most commonly recognized causes of knee replacement failures and associated pain are aseptic loosening, infection, instability, and arthrofibrosis. Proximal tibiofibular osteoarthritis has been seen as a rare cause of chronic post-TKA pain that typically presents as lateral knee pain [1].

We performed ultrasound (US)-guided proximal tibiofibular joint (PTFJ) injections in many post-TKA patients presenting with persistent nonspecific knee pain and positive PTFJ physical examination. Overall, the combined local anesthetic/steroid injections provided long-lasting pain relief that lasted for several weeks to months. Our experience demonstrates that PTFJ arthropathy may be an underdiagnosed cause of chronic post-TKA pain while the joint injections have both diagnostic and therapeutic value.

## Case presentation

We used the ultrasound-guided PTFJ injection approach in patients. Specifically, we used a 25G 1.5-inch needle guided by US to inject PTFJ, once the needle tip could be visualized entering the joint capsule, injecting a mixture of 1 ml of 0.5% bupivacaine with 1 ml of methylprednisolone (in 40 mg/ml concentration) for a total volume of 2 ml. Overall, we performed US-guided PTFJ injections in multiple patients presenting with persistent nonspecific knee pain and positive PTFJ physical exam. An overwhelming majority of the patients responded to the injections with significant reduction in pain. The response generally lasted for several weeks to several months. Here, we present three indicative cases.

Case 1

A 48-year-old male presented with progressively worsening right lateral knee pain for one year despite medications, braces, and intra-articular knee injections. He subsequently had a right total knee arthroplasty that was followed by physical therapy. The surgery did not lead to improvement in his pain. On physical examination, the patient had a linear surgical TKA scar on the anterior aspect of the knee. No redness, swelling or knee joint laxity was noted. There was global tenderness over the knee joint line as well as over the medial and lateral aspects of the joint. Pronounced tenderness was present with palpation of the fibular head. No PTFJ subluxation was present. Ultrasound examination of the PTFJ showed an intact PTFJ with no effusion. We proceeded with a bupivacaine/methylprednisolone injection into the PTFJ, which resulted in prompt reduction in pain severity with the numeric pain scale score reducing from 9 to 1. At the three-month follow-up, the patient reported continued 95% relief in pain severity, and continued ranking the pain as 1 out of 10.

Case 2

**Figure 1:**
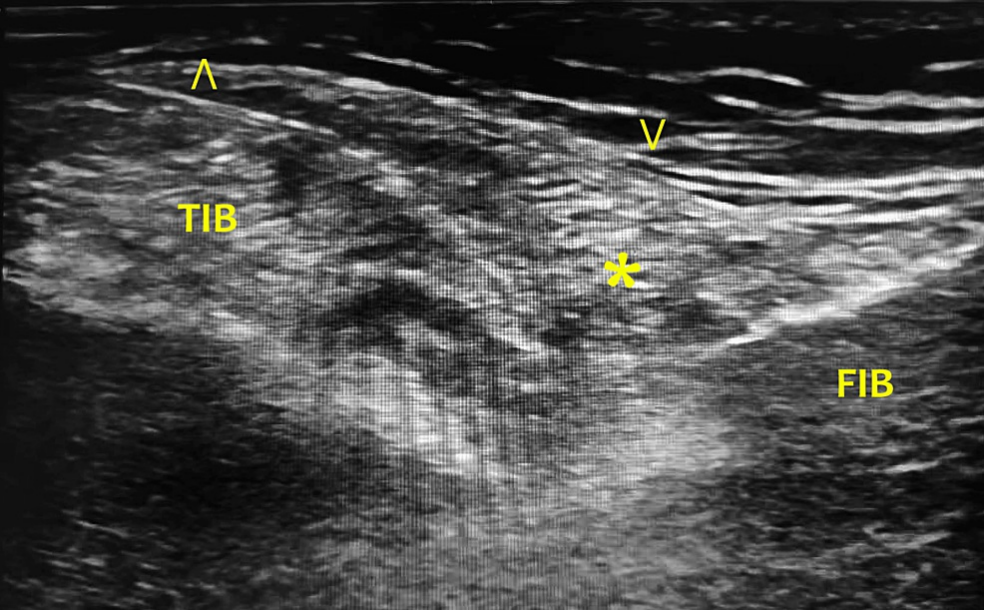
Sonographic view of in-plane PTFJ injection ˄ signifies the needle in plane, ˅ signifies the PTFJ capsule, and * signifies the injectable inside the PTFJ. FIB, fibula; TIB, tibia

A 62-year-old male presented with a six-month history of left lateral knee pain that was not alleviated by non-steroidal anti-inflammatory drugs, physical therapy, or intra-articular knee steroid injection by an orthopedic surgeon. The patient wanted a second opinion from an interventional pain specialist after he was recommended a TKA by the orthopedic surgeon. His knee MRI showed mild degenerative changes. On physical examination, no redness or swelling of the knee joint was seen. Generalized tenderness over the knee joint was noted on palpation. It was most prominent over the lateral knee aspect and PTFJ. No subluxation on manipulation of the PTFJ was appreciated. An ultrasound examination showed no effusion. We proceeded with a bupivacaine/methylprednisolone injection into the PTFJ under US guidance (Figure 1). The patient reported immediate improvement in pain with the numeric pain scale score reducing from 7 to 1. On his one-month and four-month follow-up visits, the patient continued to have substantial easing of his lateral knee pain, ranking it as 2 out of 10.

Case 3

A** **78-year-old male presented with a three-year history of chronic right knee pain one year after a TKA. The persistent pain made him unable to bear weight on his right leg. The knee pain was also associated with lower back pain, which started shortly after the knee pain. The pain was unresponsive to physical therapy, medications, and activity modification. On physical examination, a well-healed TKA scar with no signs of inflammation was noted. There was no significant laxity in the knee joint. Tenderness was present over medial and lateral knee aspects. Fibular head manipulations were associated with pain. In addition, the patient had positive hamstring tenderness preventing straight leg raise. We performed US-guided right PTFJ injection with immediate improvement in his knee pain. Two weeks later, the patient was able to bear full weight on his right leg, which he was not able to do before. His low back pain had also improved.

## Discussion

Anatomic considerations

The PTFJ is a synovial joint between the lateral tibial condyle and head of the fibula. The joint is surrounded by a dense fibrous capsule that attaches along the edges of the articular surfaces to the fibula and tibia. The capsule is strengthened by the anterior and posterior ligaments of the head of the fibula, which connect the head of the fibula with the lateral condyle of the tibia [3]. The PTFJ communicates with the knee joint in at least 10% of adults; however, the communication in up to 64% of subjects was shown in a study using MR arthrography [4]. PTFJ function is to dissipate lower leg torsional stresses as well as lateral tibial bending moments. It also plays a role in transmitting axial loads in weight-bearing.

**Figure 2:**
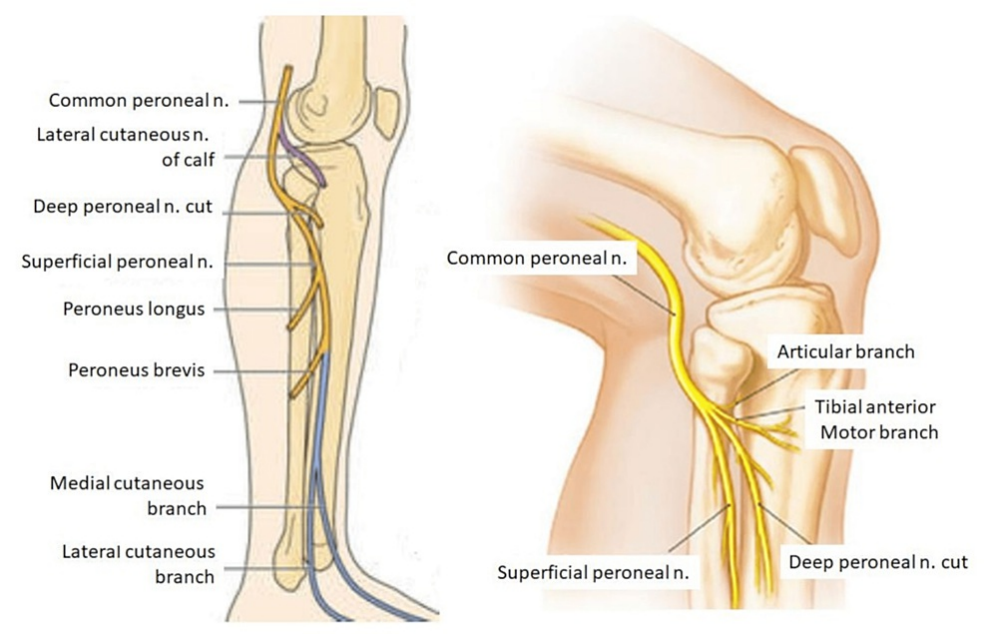
Common peroneal nerve and proximal tibiofibular joint The image is from an open access article distributed under the terms and conditions of the Creative Commons Attribution (CC BY) license [6].

The common fibular nerve wraps around the head and neck of the fibula and descends from the lateral side of the popliteal fossa (Figure 2). Distally, it continues into the initial part of the long peroneal muscle, where it divides into its two terminal branches: the superficial and deep peroneal nerves. The PTFJ is innervated by the recurrent fibular branch of the common peroneal nerve and the nerve to the popliteus (from the tibial nerve) [5].

There is a slight motion in the PTFJ with physiologic knee movement, which contributes to knee joint mechanics [7]. It is possible that an alteration in physiologic motion mechanics following TKA causes/exacerbates dysfunction of PTFJ contributing to chronic post-TKA pain.

Diagnosing PTFJ dysfunction

In general, the clinical presentation of the PTFJ pathology may vary significantly. Symptoms are nonspecific and may include lateral or anterolateral knee pain, lateral calf pain, an antalgic gait, difficulty in climbing stairs, hamstring pain or tightness, knee weakness, and symptoms of instability [8].

The PTFJ pathology may be associated with even less specific findings. For instance, in a study of 32 patients with PTFJ dysfunction, the knee pain was accompanied by low back pain in 20 patients (63.5%) [9]. There have been reports of PTFJ-associated pain radiating proximally into the region of the iliotibial band and medially into the patellofemoral joint [10]. De Franca reported a patient who had lateral knee pain, lower back pain and posterior thigh pain that were greatly diminished after PTFJ manipulation [11]. Hence, identifying PTFJ as a cause of knee pain based solely on history may be problematic.

A typical physical examination finding is lateral pain that can be elicited by fibular head palpation, PTFJ mobilization, or ankle dorsiflexion-plantar flexion in a flexed knee position. A PTFJ physical examination would be considered positive if pain and/or tenderness were elicited with the following maneuvers: (1) with the knee in a relaxed position at 45°, pressure applied to the fibular head in both anterolateral and posteromedial directions while the head is held between the thumb and index fingers, and (2) pressure to the PTFJ applied during active ankle movements [12]. Auxiliary finding of hamstring tightness is defined as follows: (1) with the knee fully extended, inability to flex the hip joint to at least 90° due to pain, or (2) during the straight leg raise test, knee is flexed to some extent due to hamstring pain [12].

The aforementioned physical exam findings should point to PTFJ dysfunction as a cause of a patient’s pain. However, sensitivity and specificity of these findings remain unclear [4,8,9]. Imaging studies, including radiography, CT, MRI, and bone scans, though useful, may not provide a definitive answer [8]. As a result, several authors have advocated the use of PTFJ injections to both diagnose and treat pain arising from this region [8,13].

PTFJ injections

There are two main approaches to injecting the PTFJ: palpation-based injection technique and ultrasound-guided injection technique. For the palpation-guided injection, the patient is seated with the knee at the right angle. The head of the fibula is identified by palpation. The joint line medial to the head of the fibula is marked. A 23G needle is inserted at the mid-point of the joint line and aimed obliquely laterally to penetrate the capsule. The solution is deposited in bolus [13].

**Figure 3:**
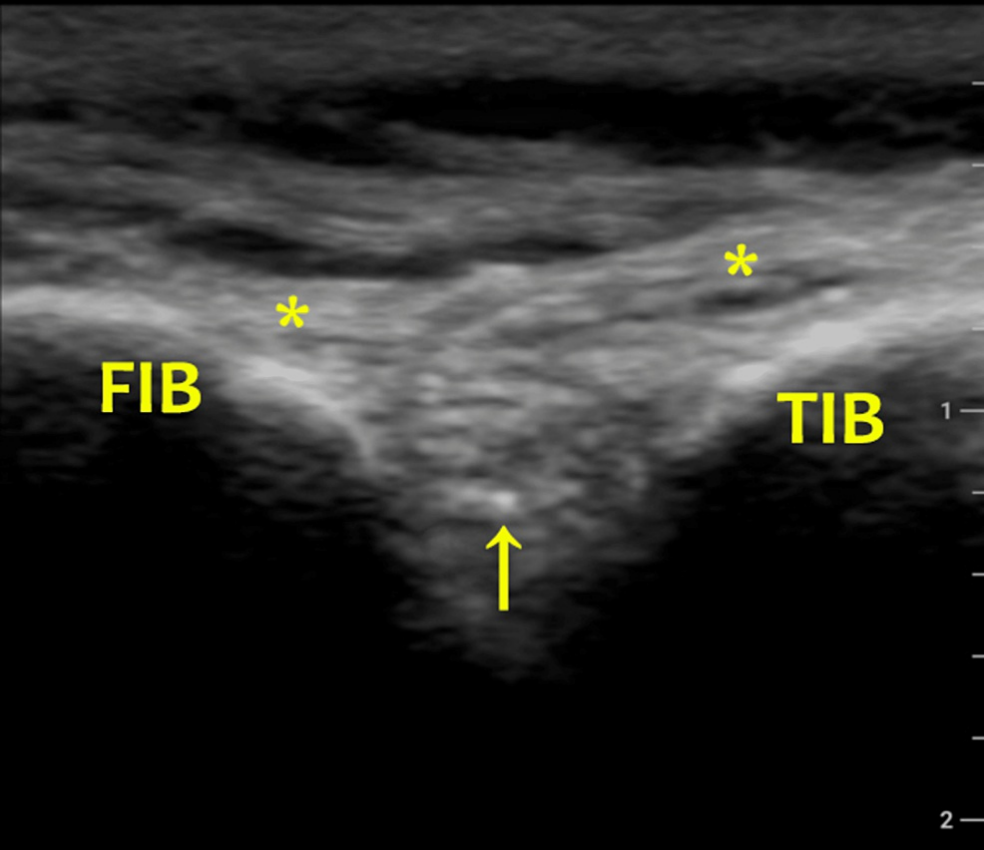
Sonographic view showing the needle tip within the PTFJ FIB, fibula; TIB, tibia; PTFJ, proximal tibiofibular joint The needle is appearing as an echogenic dot (arrow) deep into the anterior superior proximal tibiofibular ligament (asterisks). Top is superficial/anterior; bottom is deep/posterior; left is lateral; and right is medial.

In the sonographically guided PTFJ injection technique, the PTFJ injection can be performed with either in-plane or out-of-plane needle visualization. Here, we describe the out-of-plane injection technique. The patient is positioned similarly as mentioned earlier. Once the fibula head is found by palpation, the lateral end of the probe is placed on it. At this point, the medial end of the transducer is oriented toward the inferior pole of the patella so that the long axis of the transducer is perpendicular to the expected orientation of the PTFJ. Keeping the lateral end of the transducer on the fibula head, the medial end of the transducer is rotated clockwise or counterclockwise, seeking the best visualization of the PTFJ. While doing so, the anterior superior tibial ligament may serve as a reference point for the joint gap. Once the PTFJ is located, the needle is advanced under direct sonographic guidance into the joint, with the needle shaft being perpendicular to the transducer long axis (i.e. short-axis view; Figure 3) [8,14].

We used ultrasound guidance to inject the PTJF since it was demonstrated to be superior to the palpation only technique. Specifically, its superiority has been demonstrated in a study of cadaveric specimens. The study authors showed 100% versus 58% success rates with ultrasound-guided versus palpation-based techniques, respectively [8].

## Conclusions

To our knowledge, there is limited literature available on PTFJ being a source of knee pain, and this joint is usually not included in knee examination protocols. Our experience demonstrates that PTFJ injections provide considerable pain relief in patients with persistent lateral knee pain and a positive PTFJ examination. In one of the aforementioned cases, the PTFJ injection prevented a patient, who only had mild degenerative knee arthritis, from having a likely unnecessary TKA. We believe that PTFJ arthropathy may be an underdiagnosed cause of painful TKA while the joint injections have both diagnostic and therapeutic value in managing lateral knee pain. Large-scale studies would be beneficial to evaluate the injection's safety and long-term effectiveness.
